# Efficacy of the Confidential Unit Exclusion Option in Blood Donors in Tehran, Iran, Determined by Using the Nucleic Acid Testing Method in 2008 and 2009

**DOI:** 10.5812/kowsar.1735143X.778

**Published:** 2011-11-30

**Authors:** Elham Farhadi, Ahmad Gharehbaghian, Gharib Karimi, Shahram Samiee, Farzaneh Tavasolli, Yahya Salimi

**Affiliations:** 1 Blood Transfusion Research Center, High Institute for Research and Education in Transfusion Medicine, Tehran, IR Iran; 2 Medical Laboratory Sciences Department, School of Allied Medical Sciences, Shahid Beheshti University of Medical Sciences, Tehran, IR Iran; 3 Department of Epidemiology and Biostatistics, School of Public Health of Medical Sciences, Shahid Beheshti University of Medical Sciences, Tehran, IR Iran

**Keywords:** Blood Safety, Blood Donors, Iran

## Abstract

**Background:**

In recent years, the confidential unit exclusion (CUE) option has been used to increase blood safety at blood transfusion centers in several countries. The epidemiologic characteristics of diseases and demographic characteristics of patients vary in different countries; therefore, we investigated whether the CUE option is useful in Iran. In this study, we determined the prevalences of hepatitis B virus (HBV) and hepatitis C virus (HCV) in CUE-positive and CUE-negative units, as well as the efficacy of the CUE option. Objectives: The aim of this study was to evaluate the efficacy of the CUE option in reducing the prevalences of HBV and HCV in blood units.

**Patients and Methods:**

All donors were tested for the HCV antibody (anti-HCV) and hepatitis B surface antigen (HBsAg). Supplemental tests were performed to confirm the presence of viruses in the units that tested positive. In total, 2000 units (1000 CUE-positive units and 1000 CUE-negative units) were tested using the nucleic acid testing (NAT) method. The prevalence of infectious markers was estimated in all demographic subgroups.

**Results:**

The prevalences of HBV and HCV markers were higher in donors who opted for CUE than in those who did not. The CUE option had low sensitivity (21.5%) and positive predictive value (PPV; 20.9%) for the markers. Most of the donors who opted for CUE for the first time were men with low levels of education.

**Conclusions:**

The CUE option has low sensitivity and PPV, and its effectiveness in reducing the transmission of infectious diseases through window-period units is minimal. The CUE process can be continued in Iran because Iran is geographically located in a region where HBV is endemic; however, higher levels of education are necessary to make this process effective.

## 1. Background

Transfusion-transmitted infections (TTIs) are a major challenge for all organizations that conduct blood transfusions [1]. In 2007, the prevalences of hepatitis B virus (HBV), hepatitis C virus (HCV), and human imunodeficiency virus (HIV) in Iranian blood donors were 406 (per 100,000), 119 (per 100,000), and 3.9 (per 100,000), respectively [2]. In 2010, the prevalences of HBV and HCV in Iranian blood donors decreased to 0.2% and 0.07%, respectively, but the prevalence of HIV in blood donors did not change [3]. However, state reports suggest that the prevalences of HCV and HIV have been increasing in the general population. In the USA, the prevalences of HBV, HCV, and HIV were 1.5 (per 100,000), 0.3 (per 100,000), in 2007 [4] and 22.8 (per 100,000), respectively [5]. In Turkey, the prevalence of HCV (0.07%) was similar to that in Iran, but the incidences of HBV and HIV were 1.76% and 0.008%, respectively, in 2010 [6]. The prevalences of HBV and HCV were higher in blood donors in Pakistan than in Iran. The inci  nces of HBV and HCV in Pakistan were 2.4% and 3.6%, respectively [7]. Iran is located between Turkey and Pakistan, and the prevalences of HBV, HCV, and HIV were lower in the blood donors in Iran than in the blood donors in these neighboring countries. Iranian Blood Transfusion Organization (IBTO) was successful in decreasing post-transfusion infections in multitransfused patients. HCV is the most prevalent TTI in these patients. [1] The seroprevalence of HCV was 2–32% in thalassemia patients in Iran [8]; however, after the implementation of the donor screening tests for HCV, the seroprevalence in these patients was found to be 2.5% [9].

The prevalence of HCV was higher in hemodialysis and hemophilia patients than in patients with other TTIs. The seropositivities for HCV in hemodialysis and hemophilia patients were 21% and 42.5%, respectively.[1] In some regions, the seropositivity for this virus was very high. In Gilan, the prevalence of HCV antibody (anti-HCV) was 55.9% in hemodialysis patients in 2002, but it decreased to 24.8% in 2003 [1]. Successful donor selection and safety programs at the IBTO may have caused this reduction [1]. The Food and Drug Administration (FDA) recommended using the confidential unit exclusion (CUE) process for reducing TTI risk in donors who were in the window period [10]. One of the goals of the CUE process is the exclusion of blood units that may be positive for one of the transmissible diseases [11]. The CUE process was initiated in 1986.[10][12] At that time, no laboratory method for detecting HIV was present; therefore, excluding HIV-infected blood was the main goal of the CUE process. Several studies have been conducted on the efficacy of the CUE process in blood donors; in these studies, the sensitivity of CUE in detecting HIV antibody (anti-HIV) varied from 3% to 47%. The main purpose of CUE is determining highrisk donors who deny their involvement in high-risk activities when filling the screening questionnaire before donation [12]. Differences in the reports about the sensitivity of CUE may be because of the educational status of donors and because this process is relatively new in many countries.

In 1994, Korelitz et al. performed a study on the efficacy of CUE and found that the number of infectious markers such as hepatitis B surface antigen (HBsAg), anti-HCV, anti- HIV, and syphilis was 8–41 times higher in CUE-positive units than in CUE-negative units. In their study, the positive predictive value (PPV) and sensitivity of CUE were 3.5% and 2.3%, respectively [13]. In addition, they found that women used the CUE option more often than men did, and that many of the CUE-positive units had been collected from educated first-time donors [13]. A study conducted by Zou et al. in the USA showed that the CUE option has low sensitivity (3.7%), and because of this low sensitivity, 7000 blood units gad been discarded in the USA in 2001 [12]. Petersen et al. have shown that donors who used the CUE option were more likely to test positive for anti-HIV; however, because the units are infrequently excluded, the CUE process has minimal impact on blood safety [14]. In 1995, Brennan et al. reported that CUE is a useful method for routine donor selection and decreasing the incidence of TTIs [15]. O’Brien et al. have evaluated the efficacy of the CUE process by using the nucleic acid testing (NAT) method and have shown that the CUE process is not a useful safeguard layer [16].

Iran has been using the CUE process since 2003 [17]. The CUE process was designed for excluding HIV-infected blood, and the HIV window period has decreased after the use of this process;[12] however, HIV was not selected as a criterion in this study because its prevalence among blood donors in Iran was 0.005% in 2005 [1]. Because of this low prevalence of HIV, more blood units were required for this study. However, this study was a pilot study, and it was not possible to collect many blood units because there was no automated system for performing extraction and polymerase chain reaction (PCR) tests. In this study, we selected the prevalences of HBV and HCV for determining the efficacy of the CUE process. HBV was chosen because Iran is located in a region where HBV is endemic [18]; HCV was selected because its prevalence has risen from 0.12% [2] in 2007 to 0.5% [11] in 2009. Moreover, molecular methods are available for detecting HCV, thereby decreasing its window period. The evaluation of the efficacy of CUE  s necessary for continuing this process. In this study, NAT (a molecular method) was used for detecting donors in the window period. This study was performed because PCR is not used as a screening test in Iran, and the CUE option, which is used in Iran, has not been recommended by the FDA since 1992 [19].

## 2. Objectives

Some studies have reported that the NAT systems, which are used presently, may not be sensitive enough to detect all HBV-positive donors [20][21] . The objective of this study was to compare the efficacy of the CUE option to that of the NAT method in detecting window-period infections. In addition, the correlation between the prevalences of infectious markers and demographic characteristics was determined.

## 3. Patients and Methods

This study for evaluating of the efficacy of the CUE option among blood donors at the Tehran transfusion center began in 2008. In total, 353612 units of blood were donated in a year. We investigated the cause of HBV and HCV infections in blood donors. In addition, we analyzed demographic characteristics such as age, gender, and donation history. All donors were given an information sheet and were interviewed about the risk factors by a physician; they were then given some bar-coded stickers. After studying the information sheet, the donors used one of the bar-coded stickers that indicated whether they wanted their blood to be excluded. All units were tested for the necessary serologic markers at the Tehran transfusion center. HBsAg was detected using Enzygnost HBsAg 5.0 (Dade Behring), and anti-HCV was detected by ORTHO® HCV Version 3.0 ELISA Test System. Confirmatory tests for HBV were performed using neutralization tests (HBsAg confirmatory assay, Dade Behring), and anti-HCV was confirmed using the Recombinant Immunoblot 3.0 Assay (INNO-LIA™ HCV Score). We randomly collected 1000 CUE-positive blood units and 1000 CUEnegative blood units and tested them by using the NAT method for detecting HBV DNA and HCV RNA; then all results of HBV DNA, HCV RNA and other demographic data for each sample were surveyed. PCR was performed using in-house kits authenticated by the Viral Quality Control (VQC) laboratory and National Serology Reference Laboratory (NRL). The CUE forms used in Iran were analyzed and compared to the forms used in Germany.[22]

### 3.1. Statistical Analysis

The donors’ database containing their demographic characteristics, serologic markers, PCR results, and CUE option were analyzed using Statistical Package for the Social Sciences (SPSS) version 16. Chi-square test was used for comparing the data. A probability less than 0.05 (P < 0.05) and a 95% confidence interval (CI) of odds ratio OR less than 1 was considered significant. Sensitivity was defined as the proportion of CUE-positive donors among the donors identified as having an infection. The PPV was defined as the proportion of samples that tested positive for infection among the CUE-positive samples.

## 4. Results

In total, 353612 blood units were obtained from volunteers at the Tehran transfusion center between March 2008 and February 2009. The CUE option was used by 2072 (0.6%) donors. Variations in CUE according to demographic characteristics are shown in Table 1. More male donors than female donors used the CUE option (OR, 2.3; 95% CI, 1.797–2.942; P < 0.0001). Among the donors, 269541 (76.2%) were married; married donors were 0.53 times less likely than unmarried donors to use the CUE process (OR. 0.538; 95% CI, 0.482–0.601; P < 0.0001). Table 1 shows that fewer donors with higher levels of education than those with an under Diploma degree and more younger donors than older donors used the CUE option (OR, 0.55; 95% CI, 0.486–0.638; P < 0.0001). Fewer repeat donors than first-time donors used the CUE option (OR, 0.69; 95% CI, 0.623–0.783; P < 0.0001). Moreover, the frequency of the use of the CUE process by the lapsed donors was 1.5 times less than that of first-time donors (OR, 1.546; 95% CI, 1.353–1.766; P < 0.00  ).

**Table 1:** Demographic Characteristics of Donors and CUE Use

	**Donations,No.**	**Pvalue**	**Adjusted Odds Ratio**	**95% CI**
Gender				
Female	331253	0.000	1.0	1.797–2.942
Male	22359		2.300	
Age, y				
< 28	111113		1.0	
28–38	109679	0.000	0.557	0.486–0.638
> 38	132820	0.000	0.673	0.594–0.763
Education				
Under diploma	88231		1.0	
Diploma	155516	0.002	0.821	0.726–0.928
Higher than diploma	109865	0.002	0.849	0.766–0.940
Donation history				
First time	142367		1.0	
Repeat	132288	0.000	0.698	0.623–0.783
Lapsed	78957	0.000	1.546	1.353–1.766
Marital status				
Unmarried	269541		1.0	
Married	84071	0.000	0.538	0.482–0.601

### 4.1. Prevalence Rates of Infectious Disease Markers

The Tehran transfusion center had 353612 voluntary donations from March 2008 to February 2009. Of these donors, 2072 used the CUE option. Table 2 shows the prevalences of HBV and HCV. The prevalences of HBV and HCV were significantly higher in donors who used the CUE option (P < 0.05) than in those who did not. However, the CUE option had low sensitivity for detecting infection. The highest sensitivity for HCV infection was 5.5%. Furthermore, the CUE option has low PPV; the highest PPV for HBV infection was 1.5%. Donors who used the CUE option were more likely than those who did not to develop an HBV (OR, 4.63; 95% CI, 3.25–6.60; P < 0.0001) or HCV (OR, 10.13; 95% CI, 6.57–15.60; P < 0.0001) infection. Demographic subgroups of seropositive donors who used the CUE option are shown in Table 3. All donors who used the CUE option were classified according to their demographic characteristics. The sensitivity of CUE was 21.5%. Among the donors whose samples tested positive for infection, several subgroups, includi   females, elderly, and married and lapsed donors, were found to be less likely to use the CUE option. The higher the educational level of the donors, the less likely the positive donors were to use the CUE option. The use of the CUE option by infected donors decreased with increase in educational levels.

**Table 2:** The Prevalence of HBV and HCV and the Use of CUE Determined Using Serologic Tests

**ID Marker**	**Number of Donations**	**Number of Positive**	**P value**	**OR^a^**	**95% CI**	**Sensitivity**	**PPV**
HBsAg			0.000	4.63	3.25–6.60	0.0263	0.0154
No	351540	1185					
Yes	2072	32					
Anti-HCV			0.000	10.13	6.57–15.60	0.0558	0.0106
No	351540	372					
Yes	2072	22					

^a^ Abbreviation: OR, odd ratio

**Table 3:** Demographic Subgroups of Seropositive Donors That Used CUE and the Sensitivity of CUE in These Subgroups

	**Positive Units, No.**	**Sensitivity, %**	**95% CI**
Gender			
Male	83	4.31	0.0349–0.0531
Female	2	2.25	0.0062–0.0783
Age, y			
< 28	32	6.43	0.0459–0.0893
28–38	29	4.55	0.0318–0.0645
>38	24	2.73	0.0184–0.0403
Marital status			
Married	55	3.47	0.0267–0.0449
Unmarried	30	6.98	0.0493–0.0978
Education			
Under diploma	37	3.73	0.0272–0.0509
Dipl95% CIoma	31	4.18	0.0294–0.0584
Higher tdan diploma	17	6.14	0.0387–0.0961
Donation history			
First time	70	4.48	0.0356–0.0562
Repeat	7	3.33	0.0162–0.0672
Lapsed	8	3.31	0.0168–0.0639

## 5. Discussion

The CUE process was recommended by the FDA in 1986 [10][12]. The main goal of the CUE process is detecting donors who are infected but are in the window period. This process was especially designed for detecting high-risk donors who denied their involvement in high-risk activities during the predonation interview [2]. This process gives an opportunity to the high-risk donors to confidentially exclude their blood [1]. In this study, we calculated the PPV, which is based on the number of seropositive donors who understate their condition and use the CUE option correctly. Previous studies that have estimated the sensitivity of CUE have shown that it varies widely between 3% and 47% [23][24]. Different sensitivities of CUE were determined at each of the 3 centers where a single study was performed, and the sensitivities were 3%, 4%, and 37%. In this study, the sensitivity was estimated to be 21.5% and PPV to be 20.9. Although the sensitivity and PPV reported in this study are higher than those reported in the study by Zou et al. and Korelitz et al., the estimated sensitivity and PPV in this study are low for detecting window-period infections. Our data has shown that many donors who used the CUE option had positive test results; however, the differences between the test results of the CUE-positive and CUE-negative donors were not significant. Our findings were confirmed in a study by Omid Khoda et al. They showed that more CUE-positive donors than CUE-negative donors had positive test results of infection [25] . Kean et al. have proved that the low sensitivity and PPV of the CUE option could be attributed to errors in selecting the CUE option by the donors. They reported that most of the donors that opted for CUE had said “I wasn’t paying attention” [26]. Another reason might be the donors’ education, which may not have been high enough. Yet another reason may be related to the CUE form. According to Sumnig’s study, the CUE forms need certain characteristics such as using pictures instead of text and a colored note in  ead of black note. The CUE form has not yet been evaluated in Iran; however, we believe that the evaluation of this form may be effective for increasing the sensitivity and PPV of the CUE option [22] . Out of the 353612 donors in this study, 2072 (0.6%) used the CUE, and among these 2072 donors, about 21% tested positive for infection; therefore, we believe that the donors do not understand the usage of the CUE option. Table 1 shows that more donors with low levels of education than those with high levels of education used the CUE option. More first-time donors than repeat donors used the CUE option (OR, 0.698; 95% CI, 0.623–0.783; P < 0.0001). This finding confirms our hypothesis because repeat donors are more familiar with blood transfusion. In this study, more male donors than female donors used the CUE. Women in Iran are reluctant to donate blood because they are afraid of becoming anemic and contacting infections [27]. In this study, 2000 blood units (1000 CUE-positive units and 1000 CUE-negative units)  ere tested for HBV DNA and HCV DNA by using the NAT method. There were several infected units in both the groups, all of which were seropositive for either HBV or HCV; consequently, the NAT method was not able to detect the blood units that were in the window period. The findings of the NAT method are shown in Table 4. The NAT method decreases the window period; however, this method does not eliminate the window period. Therefore, such tests cannot eliminate the risk of transmissible diseases. The number of blood units tested using the NAT method was less, and these tests need to be repeated with a large number of units. PCR was performed by using in-house kits, none of which was automated. Furthermore, genome extraction was performed manually; therefore, we were unable to test more blood units by using the NAT method. Moreover, the NAT method cannot significantly change its window period. Analysis of the obtained data, suggests that the CUE option can be used as an extra safeguard, particularly in Iran beca  e it is located in a region where HBV is endemic. In conclusion, higher education among the people of Iran is necessary for continuation of the CUE process. The format of the CUE forms may be the cause of its low efficacy; thus, a survey on Iran’s CUE forms is necessary, and further studies need to be performed for the evaluation of the CUE option.

**Table 4:** The Prevalences of HBV and HCV and the Use of CUE in the NAT Method in 2000 Units of Blood

**ID Marker**	**Number of Donations**	**Positive Findings, No.**	**P value**	**OR^a^**	**95% CI**	**Sensitivity**	**PPV**
HBV-NAT			0.0583	6.03	0.728–277.6	0.08571	0.006
No	1000	0					
Yes	1000	6					
HCV-NAT			0.3168	3	0.240–157.9	0.75	0.003
No	1000	0					
Yes	1000	4					

^a^ Abbreviation: OR, odd ratio
